# Supplementary material for: Sources of predictive information in dynamical neural networks
Source: Sci Rep. 2020 Oct 9;10:16901. doi: 10.1038/s41598-020-73380-x (PMC7547683; doi:10.1038/s41598-020-73380-x)
Supplement: Supplementary file 1 — Supplementary Information. [file 41598_2020_73380_MOESM1_ESM.pdf]

Sources of predictive information in dynamical neural networks

Madhavun Candadai, Eduardo J. Izquierdo

Supplementary Figures

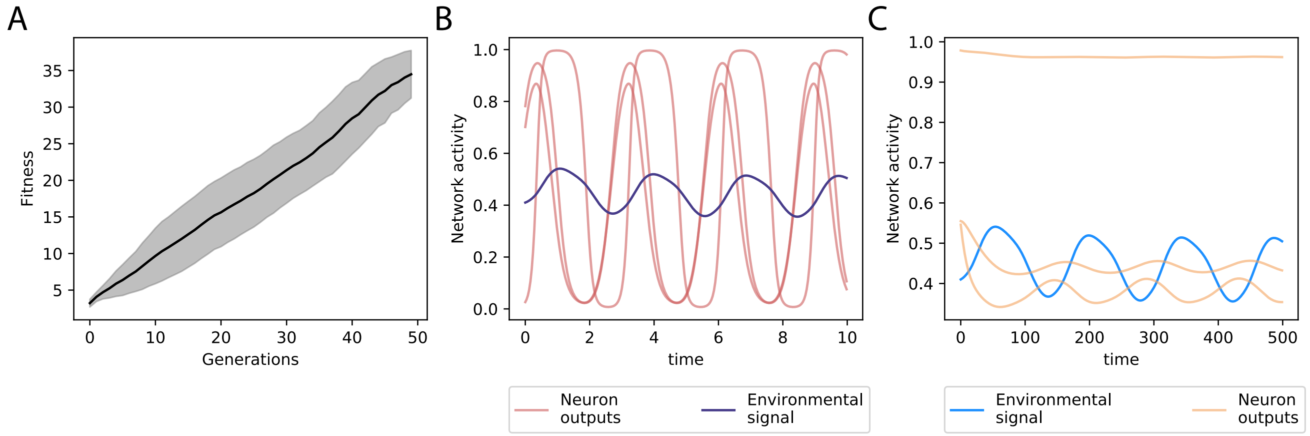

Figure S1. Optimization and neural traces of CPG and PP. [A] Fitness over time for 100 valid runs of optimizing a CPG model. Only runs that achieved a fitness greater than 30 were deemed valid. [B] Neural traces from one trial of the best CPG demonstrating that all neurons (red) as well as the neural network output (blue) oscillate. [C] Neural traces (orange) when the output from the CPG shown in panel B was fed to a random neural network in the PP condition demonstrating input driven oscillation in the random neural network.

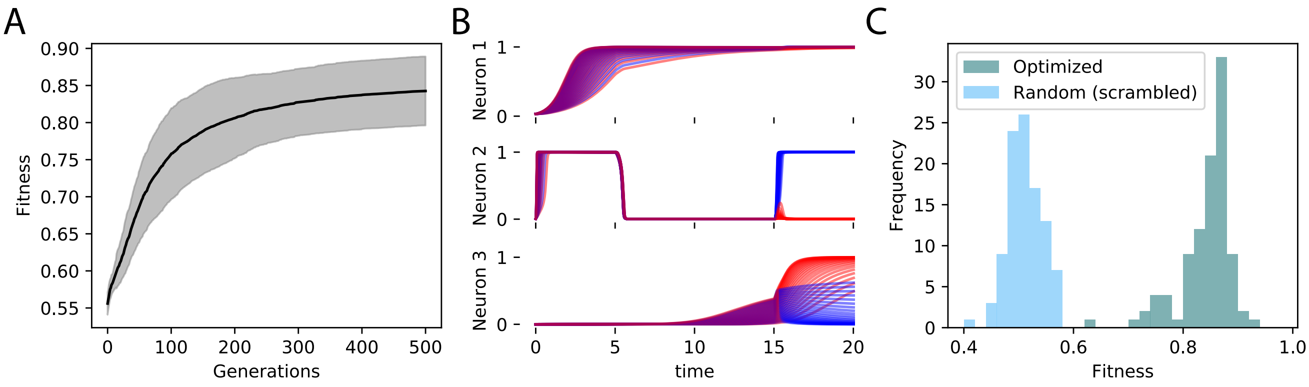

Figure S2. Optimizing neural networks to perform relational categorization. [A] 100 independent runs all converged to near-perfect performance with deviation from a perfect score only due to small deviations from expected output and not mis-categorization. [B] Neural activity in the CTRNN of the best optimized agent over 35 trials where probe was larger than the cue (red) and 35 trials where the probe was lesser than the cue (blue). [C] Neural networks whose weights and time-constants were scrambled lost their ability to perform the task.

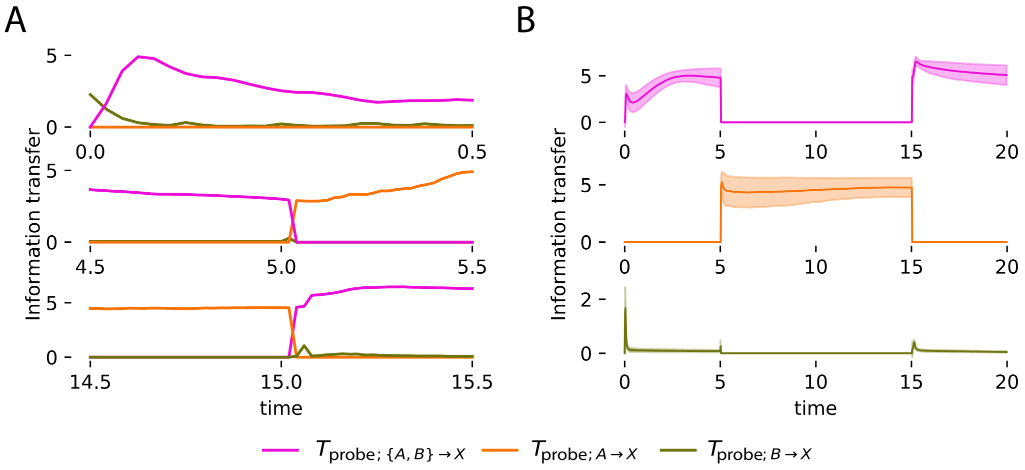

Figure S3. Predictive information source dynamics is consistent and similar with information about the probe. [A] At the start of the cue stage (top), information about the probe arrives from the environment (green) as the cue is provided, and becomes redundant as the cue is encoded (pink). Towards the start of the delay stage (middle), the neural network becomes the source of information about the probe (orange) as it retains information about the cue, and since the environment ceases to provide that information. As the probe is provided (bottom), the environment once again becomes a source of information in addition to the neural network and they are both redundantly sources of information (pink) [B] Predictive information source dynamics are consistent across all 100 optimized neural networks during all three stages of the task. Their mean value is shown in bold and the shaded region represents one standard deviation around it.

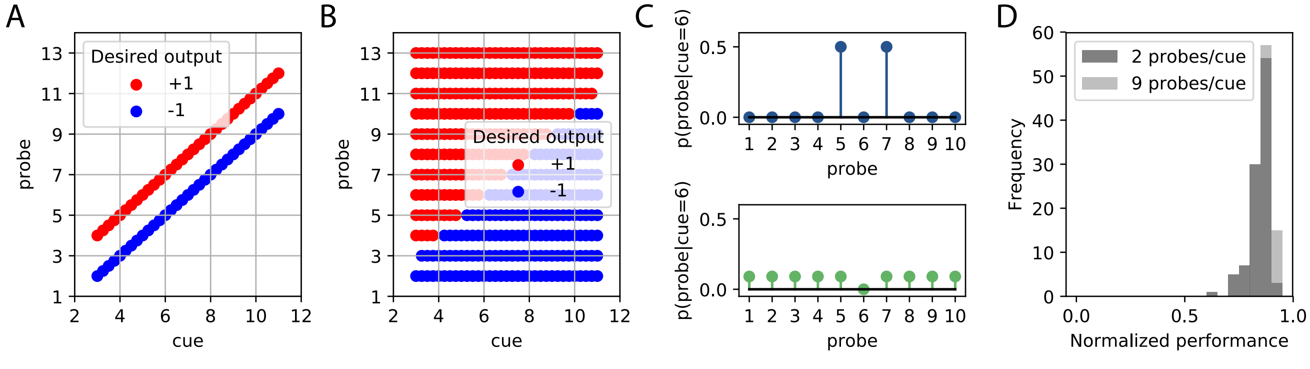

Figure S4. Different environmental structures within the relational categorization task [A] Relational categorization task with highly structured stimuli; for each cue probe is one of two possible values. [B] Relational categorization task with minimal structure in stimuli; probe can be one of 9 values for a given cue. [C] Conditional probability of probes given a cue for environmental structure shown in panel A, demonstrating the significant reduction in uncertainty of the probe given the cue. [D] Conditional probability of probe values given a cue under the environmental structure in panel B shows that probe values still have a nearly uniform distribution, and hence very less reduction in uncertainty. [E] Neural networks optimized to perform under the distribution shown in panel A perform just as well under the distribution shown in panel B.

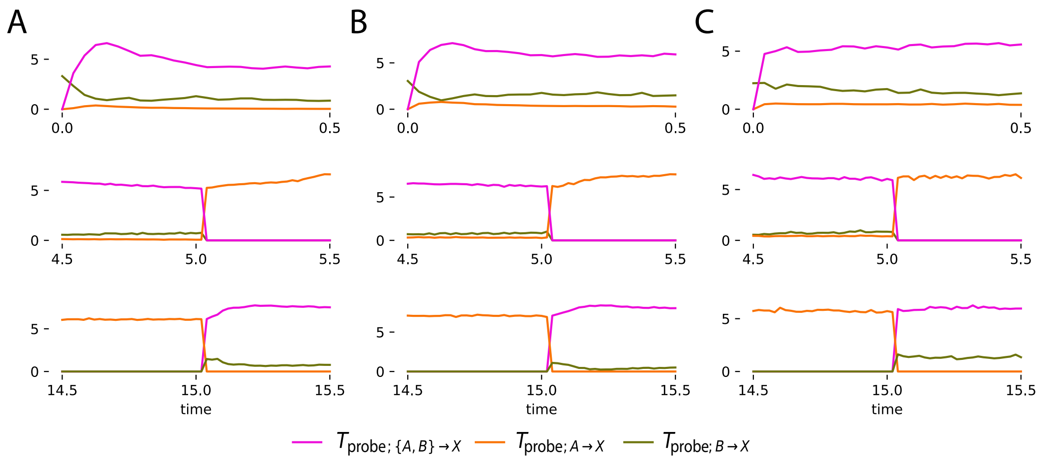

Figure S5. Inferring the source of predictive information is robust to zero-mean Gaussian noise with standard deviation [A] 0.01, [B] 0.05 and [C] 0.1. Results are qualitatively similar to results from fig. S3A for cue (top row), delay (middle row) and probe (bottom row) stages of the task.

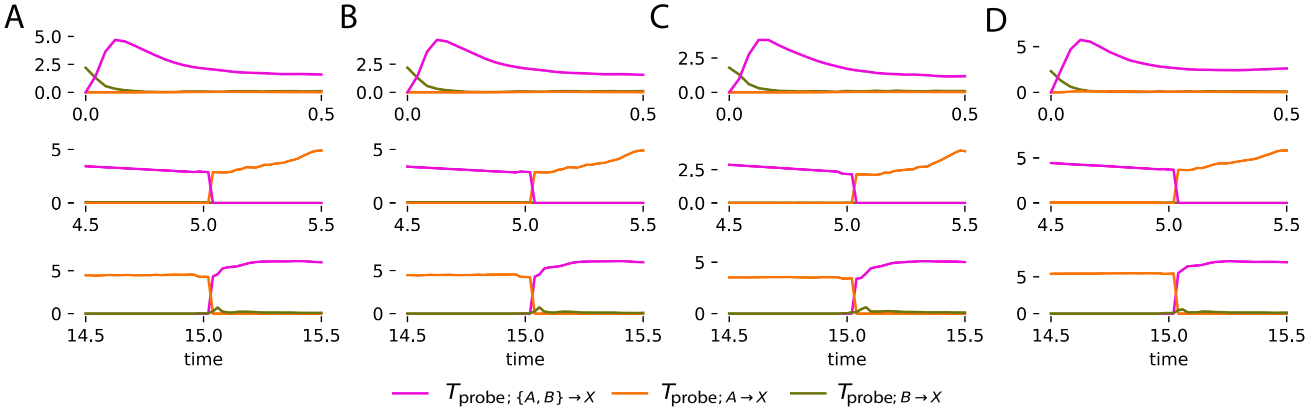

Figure S6. Inferring the source of predictive information with different binning and shifted-histograms. Results are qualitatively similar to results from fig. S3A after changing [A] number of shifted bins to 3 [b] number of shifted bins to 11 [C] number of bins per dimension to 50 and [D] number of bins per dimension to 200, for cue (top row), delay (middle row) and probe (bottom row) stages of the task.
